# Assessing the Effect of Polyethylene Microplastics in the Freshwater Leech *Erpobdella johanssoni* (Annelida, Hirudinida) Through Integrated Biomarkers and Histopathological Analysis

**DOI:** 10.3390/ani15101417

**Published:** 2025-05-14

**Authors:** Raja Ben Ahmed, Ichrak Khaled, Tahani El Ayari, Issam Saidi, Abdel Halim Harrath

**Affiliations:** 1 Ecology, Biology and Physiology of Aquatic Organisms Laboratory, Department of Biology, Faculty of Sciences of Tunis, University of Tunis El Manar, Tunis 2092, Tunisia; raja_benahmed@yahoo.fr; 2 Laboratory of Biotechnology and Biomonitoring of the Environment and Oasis Ecosystems (LBBEEO), Faculty of Sciences of Gafsa, University of Gafsa, Gafsa 2112, Tunisia; ichrakk@gmail.com (I.K.); issam.saidi@gmail.com (I.S.); 3 Group of Fundamental and Applied Malacology (LEB/GFAM), Laboratory of Environment Biomonitoring, Faculty of Sciences of Bizerte, University of Carthage, 7021 Zarzouna, Bizerte 1054, Tunisia; tahaniayari@yahoo.fr; 4 Faculty of Sciences of Gafsa, University of Gafsa, Gafsa 2112, Tunisia; 5 Department of Zoology, College of Sciences, King Saud University, Riyadh 11451, Saudi Arabia

**Keywords:** plastic pollution, toxicity assessment, oxidative stress, lipid peroxidation, histopathology, Hirudinea

## Abstract

Microplastics (MPs) are contaminants of emerging concern, and the study of their effects on several species, especially freshwater organisms, has not been exhausted. *Erpobdella johanssoni*, a freshwater leech, was used as a model in this study for the assessment of the potentially toxic effects of polyethylene microplastics (PE-MPs) 40–48 µm in size under controlled laboratory conditions. PE-MP toxicity was assessed in the examined leech using four increasing concentrations (1, 10, 100, and 1000 µg/L) during an exposure period of 7 days. Oxidative damage was detected through the increase in malondialdehyde (MDA) levels, reflecting the occurrence of lipid peroxidation. The activities of enzymes involved in the antioxidant response, such as catalase (CAT), superoxide dismutase (SOD), and glutathione peroxidase (GPx), increased. Furthermore, the histopathology of the body wall, muscle cells, botryoidal cells, and ovaries was assessed to understand and evaluate the acute toxicity of PE-MPs. The histopathological changes detected in PE-MP-treated leeches varied in a concentration-dependent manner. Overall, this research indicated that even at environmentally relevant concentrations, PE-MPs had biological effects on the studied leeches and, consequently, on the freshwater invertebrate clade. This finding could provide novel insights into the effects of plastic pollution on freshwater ecosystems, particularly on invertebrate health and biodiversity.

## Simple Summary

Microplastics are tiny pieces of plastic found in the environment, especially in water. In this study, we investigated how exposure to polyethylene microplastics affects freshwater leeches. We found that even low concentrations of microplastics caused damage to the leeches’ cells and tissues. This research highlights the harmful effects of plastic pollution on aquatic life and emphasizes the need to address plastic waste in our ecosystems.

## 1. Introduction

Plastics, generally categorized into macroplastics and microplastics, have achieved global commercialization owing to their affordability, ease of manufacturing, lightweight nature, and long-lasting properties [1]. Additionally, plastics serve as an effective thermal and electrical insulator [2]. However, their widespread use has led to significant environmental concerns, particularly regarding microplastic (MP) pollution in aquatic ecosystems.

MPs are synthetic organic polymer particles less than 5 mm in size [3]. In recent years, MP release has increased substantially, especially with the increasing incidence of coronavirus disease 2019 (COVID-19) [4,5,6]. Discarded facial masks are estimated to contribute to the release of 1370 trillion MPs into the marine environment by 2020 [7]. Once in the aquatic environment, MPs tend to accumulate in sediments and are thereafter ingested by benthic organisms as a consequence of their nonbiodegradability [8]. Furthermore, the small size, long-lasting nature, and widespread distribution of microplastics through water have frequently been associated with various harmful effects on a wide range of organisms [9,10]. These effects can include tissue damage, reduced reproductive capabilities, and increased susceptibility to oxidative stress [11,12]. Most of the published studies on MP pollution have focused on determining polymer amounts and types, as well as on assessment of the environmental risk that MPs pose for marine organisms [13,14,15]. However, research into the impacts of microplastics on freshwater species remains limited [16], particularly concerning invertebrates such as leeches. Currently, freshwater ecosystems serve as important environments for many species, providing essential resources and supporting key ecological functions. However, they are facing significant microplastic pollution originating from sources such as wastewater, terrestrial runoff, and industrial discharge [17,18]. Recent data indicate that inland freshwater ecosystems often bear a more substantial burden of microplastic contamination compared with marine environments [19,20]. These records have surpassed previous maximum density levels, reaching a staggering concentration of 1, 146, 418.36 items/m^3^ [21]. Earlier studies have reported the contamination of karst aquifers by MPs in a number of countries, including the United States, Germany, and Italy [22,23]. This finding has raised awareness about the hazardous occurrence of MPs in freshwater ecosystems [8]. Most of the detected MPs in surface water, sediments, and groundwater have been identified as polypropylene (PP), polyethylene (PE), polystyrene (PS), and polyethylene terephthalate (PET) [24,25,26]. In Tunisia, the MP concentration is estimated to be 6920 items kg^−1^ dry weight in sediments and 18,000 items kg^−1^ in freshwater and marine ecosystems, respectively [27,28]. Furthermore, a study in the Seine River, France, found an average of 100.6 ± 99.9 fibers/m^3^ in the 50–5000 μm size range. Similarly, research in the Teltow Canal, Germany, reported 7.86 ± 7.26 MPs/L in the 450–5000 μm range. These findings underscore the widespread occurrence of MPs in freshwater environments and highlight the need for further research to assess their distribution, sources, and potential impacts (for details, see Table 1).

Assessing microplastic exposure in organisms within their natural environments poses significant challenges. This is due to the heterogeneous distribution of MPs and the difficulty in distinguishing their effects from those of other pollutants [32]. Consequently, laboratory-based studies are necessary to elucidate the potential impacts of microplastic exposure [33]. While extensive studies have been conducted on marine species, freshwater bioindicator organisms remain underexplored in this context.

As mentioned above, microplastic bioaccumulation and its effects have been studied mainly on marine species such as edible mollusks (Mediterranean mussel (*Mytilus galloprovincialis*), carpet clam (*Ruditapes decussata*), common cuttlefish (*Sepia officinalis*), horned murex (*Hexaplex trunculus*), brown murex (*Bolinus brandaris*), and Pacific oyster (*Crassostrea gigas*)) and fishes (European bass (*Dicentrarchus labrax*), painted comber (*Serranus scriba*), white sea bream (*Sarpa salpa*), and golden gray mullet (*Liza aurata*)) [34,35,36,37,38]. Nevertheless, to monitor aquatic pollution, it is essential to identify new bioassay organisms that can serve as reliable indicators of microplastic toxicity in freshwater environments.

Leeches have proven to be valuable bioindicators in ecotoxicology due to their sensitivity to various pollutants and their widespread presence in freshwater ecosystems [39,40,41]. Compared with research into insects, mollusks, and other invertebrate groups, relatively little published research is available on the use of leeches in toxicity tests. Species belonging to the genus *Erpobdella* are abundant and are easily collected and handled [42]. For *Erpobdella johanssoni*, the ecotoxic effects of benzene, toluene, ethyl benzene, and xylene (BTEX) on the genital organs were investigated [43,44]. Furthermore, its anatomy has been well demonstrated [45]. To our knowledge, the present study is the first to investigate the effects of polyethylene microplastics (PE-MPs) ranging from 40 to 48 µm in size on this freshwater leech. Polyethylene microplastics are the most commonly produced microplastics worldwide and are consequently the most commonly detected in the environment [30,46]. Hence, this study aimed to investigate the toxicity of PE-MPs on the freshwater leech *Erpobdella johanssoni*. The oxidative stress status was evaluated using enzymatic antioxidant responses (catalase (CAT), superoxide dismutase (SOD), and glutathione peroxidase (GPx) activities) and a nonenzymatic antioxidant response (malondialdehyde (MDA) levels). Histopathological analysis was performed to determine the effects of the four tested concentrations of PE-MPs on the ovary, muscle cells, botryoidal cells, and body wall structure. As is commonly understood, the epidermis serves as the primary barrier against pathogens by providing physical and immunological defense [47,48]. Similarly, mucus plays a significant role in protecting against harmful organisms [49]. Moreover, microplastic reproductive toxicity was reported to be closely related to oxidative damage [50].

## 2. Materials and Methods

### 2.1. Experimental Exposure to PE-MPs

**Figure 1:**
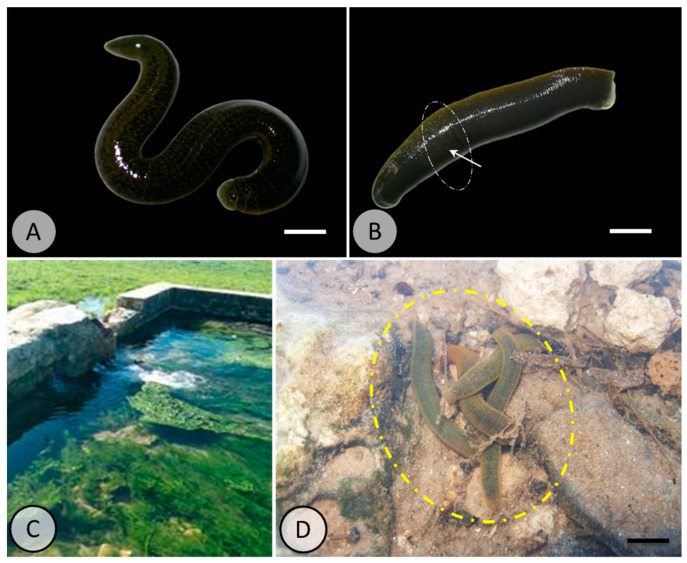
*Erpobdella johanssoni* (**A**): dorsal face, scale bar = 1.6 cm; (**B**): ventral face. Note that the clitellum of these specimens is visible, indicating that they are mature. Moreover, the male gonopore is indicated by the arrow. Scale bar = 1.8 cm. (**C**): sampling site: “Aïn Nfaja”, 24 km before Sejnen, Mateur (37°02′291″ N, 09°26′219″ E), Bizerte governorate. (**D**): the circle indicates Erpobdella specimens found free-living under rocks at the collection site. Scale bar = 1.4 cm.

Healthy adult specimens of *Erpobdella johanssoni* of similar sizes were collected in January 2023 from Ain nafja (N37.02291/E09.26219), Sejnane, Bizerte (northern Tunisia) (Figure 1). They were transferred directly to the laboratory in aerated tanks containing source water. Acclimation was carried out for 7 days in an aquarium containing 10 L of dechlorinated tap water under normal conditions (temperature: 18 ± 0.1 °C; photoperiod: 12 h light/dark cycle). After acclimation, the *E. johanssoni* specimens were randomly divided into four groups; each group consisted of 12 individuals. Each group was run in triplicate. A control group of 12 specimens was kept in clean water. The exposure to polyethylene microplastics (PE-MPs) (40 μm and 48 μm in size, selected based on their frequent occurrence in freshwater environments, as reported in previous studies) lasted 7 days. The PE-MP concentrations used were as follows: 1, 10, 100, and 1000 µg/L. PE-MP concentrations were chosen based on previous studies, and the first three concentrations were reported as environmentally relevant concentrations [51]. The latter was considered a high concentration [52]. During the experiments, no signs of mortality were observed. Polyethylene microplastics were purchased from Sigma-Aldrich (St. Louis, MA, USA). PE-MPs were prepared from a stock solution [1 g/L]; 100 mg of microplastic powder was dissolved in 100 mL of ultrapure water, and the mixture was then homogenized for 30 min using a magnetic stirrer.

### 2.2. Measurement of Biochemical Parameters

A total of 10 leeches per condition were homogenized in 0.3 mL of Tris saline buffer (TSB). The homogenate was then centrifuged at 12,000 rpm for 20 min at 4 °C. The supernatants were transferred to a new Eppendorf tube and kept at −80 °C for further use. Total protein concentrations were determined using Bradford reagent, with bovine serum albumin as a standard [53]. All enzyme preparations were carried out on ice.

The catalase (CAT) activity was determined by following the method of Aebi et al. [54], which involves measuring hydrogen peroxide (H_2_O_2_) decomposition at 240 nm using an extinction coefficient of 0.043 mM^−1^ cm^−1^. The specific activity of the enzyme was expressed as µmol H_2_O_2_/min/mg of protein.

Superoxide dismutase (SOD) activity was assayed as described in [55]. The enzyme amount was estimated through the estimation of nitro blue tetrazolium (NBT) inhibition at 25 °C. The enzyme activity was expressed as U mg^−1^ protein.

Glutathione peroxidase (GPx) activity was assessed using the method of Flohé and Günzler [56]. This method is based on the assessment of GSH oxidation by GPx in the presence of 5,5′-dithiobis-(2-nitrobenzoic acid) (DTNB) (10 mM) and GSH as a substrate. GPx activity was measured at 412 nm, and the results are expressed as nmol GSH/min/mg protein.

Malondialdehyde (MDA) levels were determined using the method described by Gutteridge and Halliwell [57], with thiobarbituric acid as a reactive substance (TBARS). The optical density was measured at 535 nm, and the amount of MDA was expressed in nmol mg^−1^ protein. For each measurement, three replicates were performed to ensure statistical reliability.

The Bradford reagent, bovine albumin, thiobarbituric acid, NBT, DTNB, and GSH were purchased from Sigma-Aldrich, St. Louis, MO, USA.

### 2.3. Behavioral Assessment

Leeches were placed in glass dishes containing experimental solutions to evaluate behavioral alterations through direct observation. Throughout and after exposure to polyethylene microplastics (PE-MPs), changes in body shape, sucker positioning, and swimming activity were monitored. The assessed parameters included body contractions, coiling, abnormal sucker positioning, and overall mobility. To evaluate recovery, leeches were transferred to microplastic-free water, and their behavioral responses were observed over the following hours.

### 2.4. Histological Staining

At the end of the experiment, two specimens from each condition were selected to ensure a representative analysis and were then fixed in Bouin’s solution for 24 h. Histological sections were prepared from the middle part of the body, which contains all the key organs, including the gonads. Two tissue samples per condition were then washed with a continual flow of water and finally preserved in 70% ethanol. The ethanol was changed several times until the samples became free from Bouin’s solution. For the preparation of the paraffin tissue blocks, an increasing volume of ethanol from 80% to 100% was applied for 1 h each, and the final bath was toluene. Five-micron-thick sections were cut using a Leica microtome and later stained with hematoxylin and eosin. To reduce bias, multiple sections were analyzed per specimen, ensuring a thorough evaluation of all key structures. Photographs were taken with a Leica Dm 500 light microscope (Leica Microsystems, Wetzlar, Germany).

### 2.5. Statistical Analysis

The data are expressed as the mean ± standard error of the mean (SEM). The normality of the data was first assessed using the Shapiro–Wilk test, and the homogeneity of variances was tested using Levene’s test. The statistical significance of the differences in enzyme activity between the unexposed and exposed groups was first examined via one-way analysis of variance (ANOVA) in GraphPad Prism version 9 software. Significant differences were subsequently determined using Tukey’s multiple comparison test. A probability level of less than 0.05 was considered significant (95% confidence interval).

## 3. Results

### 3.1. Histopathological Analysis

#### 3.1.1. Alterations in the Body Wall

**Figure 2:**
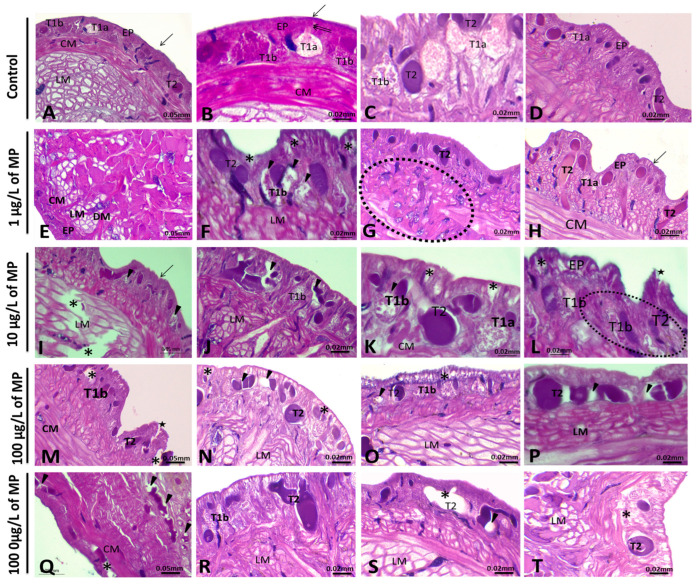
Morphological and optical microscopic analyses of *E. johanssoni* body wall sections after exposure to different concentrations of MPs. The general view of the body wall of untreated leeches (**A**–**D**) shows that the epidermis with epithelial cells (EP), cuticle (arrow), and rounded inactive secretory cells are located under the epithelium: pear-shaped cell type 1 (T1a (light-stained) and T1b (dark-stained)) and type 2 (T2). The double arrow in (**B**) indicates the duct of pear-shaped cell type 1 (T1a). Circular muscles (CMs) and longitudinal muscles (LM) are also visible. In the experimental group (1, 10, and 100 µg/L MP), all types of mucus cells appeared active and thus more elongated (i.e., see circle in (**G**,**L**)). Compared with those in the control group, there were also numerous secretory cells migrating toward the subepithelial area (encircled). The revitalization of the secretory cells essential for secretion, thereby enabling a fresh influx of mucus, is also possible. Furthermore, in the treated group, several histopathological alterations were noted: vacuolar degeneration in the epidermis (asterisk), deterioration and changes in the morphology of all secretory cell types (arrowheads), and detachment of the cuticle (stars). Circular cells (CM), dorsoventral muscles (DM), and longitudinal muscles (LM) are also marked (H&E: hematoxylin and eosin staining; magnification ×40; scale bars indicated in each panel).

In the untreated leeches, the body wall exhibited a distinctive structure. A thin layer of cuticle forms the outer surface of the epidermis, beneath which lies a layer of large columnar epithelial cells in direct proximity to the cuticle. Additionally, three types of secretory cells were scattered among the epithelial cells (Figure 2). Light-stained (type T1a) and dark-stained (type T1b) cells were found close to the epidermis. They are rounded cells and contain granules. Some of them appeared to be pear-shaped. They have a large base with a thin, long, narrow duct connecting it to the surface (Figure 2B,C). In contrast, T2 secretory cells reach the inner parts of the body wall, especially around the muscle layer (Figure 2A,D). Furthermore, it is important to emphasize that in the body walls of normal, untreated adult animals, blood vessels are practically absent (Figure 2A–D).

**Figure 3:**
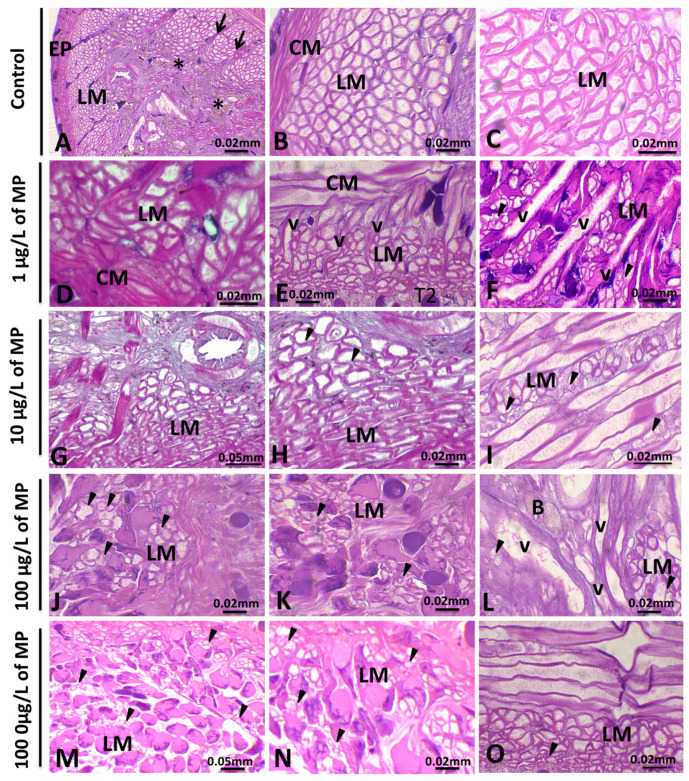
Histological analysis of the body wall muscles of leeches exposed to PE-MPs. Hematoxylin and eosin staining of longitudinal sections from untreated and PE-MP-treated leeches (**A**–**O**). In untreated leeches (**A**–**C**), muscle fibers appeared essentially regular and well organized. Note that three types of muscle fibers are observed: circular cells (CM), dorsoventral muscles (arrows), and longitudinal muscles (LM). In (**A**), we note that longitudinal muscle (LM) fibers are organized in the dorsoventral muscle (DM) field. (*) indicates botryoidal tissue in the subepithelial area; (EP) marks the epidermis. After 7 days of plastic exposure (**D**–**O**), there was a visible increase in new vessels (v) in the subepithelial area between muscles. Additionally, the longitudinal muscles appear irregular and disorganized, suggesting an impact on muscle function and cell integrity (arrowheads); B: botryoidal tissue.

**Figure 4:**
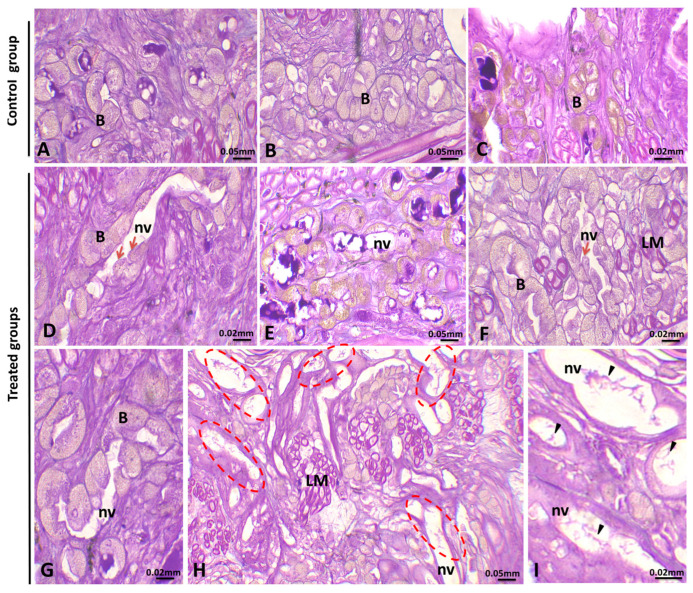
Histological analysis of botryoidal tissue morphology in untreated and PE-MP-exposed *Erpobdella johanssoni*. In untreated specimens (**A**–**C**), the nonactivated botryoidal tissue consisted of stable, compact cords containing clusters of large, rounded granular cells. In exposed animals (**D**–**I**), botryoidal cells (B) underwent significant shape modifications, including thinning and flattening, thus forming a half-moon-shaped structure, and thus were considered activated. The central lumen of the new vessels (nvs) is lined by botryoidal tissue cells (arrows). In addition, numerous circulating precursor-like cells (circle in (**H**) and arrowheads in (**I**)) are visible within the bloodstream. LM: Longitudinal muscles are also seen.

Leeches exposed to PE-MPs exhibited various histopathological changes, which were consistently observed in all exposed specimens. Notable changes included cuticle degeneration, the appearance of degeneration vacuoles in the epidermis, and a change in the morphology of the secretory cell type, which appeared more elongated than that in the control group (Figure 2E–T). The number of secretory cells (both type 1 and type 2) increased with alterations in the morphology of leeches exposed to higher doses (100 and 1000 μg/L of PE-MPs). Additionally, these leeches displayed deformation of the cuticle and epidermis, characterized by the presence of large degeneration vacuoles (Figure 2E–T). Additionally, several vessels found between the gut and the body wall were observed, with indications suggesting the likely presence of hematopoietic precursor cells originating from botryoidal tissues (Figure 3F,L and Figure 4H,I). These changes occurred in a dose-dependent manner.

#### 3.1.2. Alterations in Muscle Cells

In untreated leeches, the internal dermis consists of several layers of muscle. It contains a relatively thin outer layer of circular muscles, a thin layer of oblique muscles, and a thick inner layer of longitudinal muscles. Moreover, a dorsoventral layer of muscles is present (Figure 3). Within the control group, muscle fibers exhibited a consistent and regularly arranged pattern characterized by a uniform and orderly appearance. Conversely, leeches treated with and exposed to microplastics, especially at high doses, displayed muscle fibers with pronounced irregularities and disorganization (Figure 3G–J). Particular attention was paid to alterations in the longitudinal muscle. These changes were observed in a dose-dependent manner, with greater irregularities and disorganization at higher concentrations of microplastics.

#### 3.1.3. Alterations in the Botryoidal Tissue

In the space between the musculocutaneous region and the digestive tube, there is a thick layer of loose connective tissue containing botryoidal tissue (Figure 4). In untreated leeches, nonactivated botryoidal cell clusters typically contained large, oval/rounded granular cells densely packed in a rope-like structure, occasionally forming small lacunae (Figure 4A–C). However, in all experimental groups (1, 10, 100, and 1000 µg/L of PE-MPs), a transition in the architecture of botryoidal tissue was observed, shifting from a clustered, cord-like arrangement to half-moon-shaped cells with a hollow, tubular structure (Figure 4D–I). At this stage, they were considered activated. Moreover, the alterations showed a concentration-related trend, with histopathological changes appearing more frequently and extensively at higher exposure levels.

#### 3.1.4. Alteration in the Ovary

**Figure 5:**
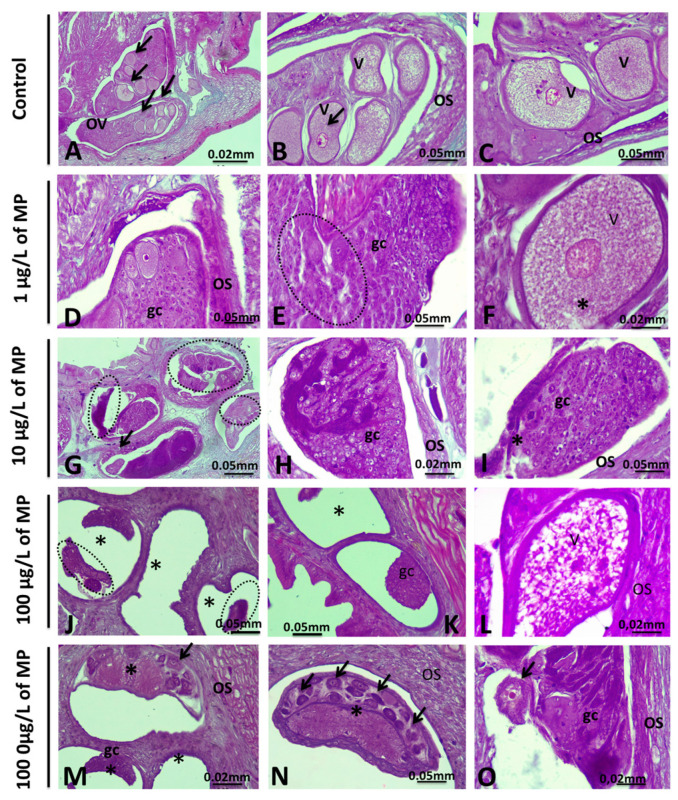
Histological analysis of ovisacs of *Erpobdella johanssoni* after exposure to polyethylene microplastics (PE-MPs). (**A**–**C**): Hematoxylin and eosin (H&E) staining of paraffin-embedded ovisac (os) sections of *Erpobdella johanssoni* from the control group showing normal structure, as evidenced by the well-organized ovary cord (ov) and by the normal morphology of both developing oocyte (arrows) and vitellogenic oocyte (v). (**D**–**F**): Effect of 1 µg/L of PE-MP exposure on *E. johanssoni* ovisacs (os) indicating a progressive alteration of the germ cell cysts (gc) forming the ovary cord integrity (delineated by ellipses). Moreover, the ooplasm of the vitellogenic oocyte (v) is affected (asterisk). (**G**–**I**): Effect of 10 µg/L of PE-MP exposure on *E. johanssoni* ovaries. Note the increased number of abnormally growing vitellogenic oocytes (ellipses, arrows, and asterisks). (**J**–**O**): Ovaries from animals treated with 100 and 1000 µg/L of PE-MP_S_. The almost complete degeneration of the normal architecture of the ovary cord (asterisks and ellipses) and the normal morphology of vitellogenic oocytes (arrows) are shown. (H&E ×40).

*E. johanssoni* ovaries showed several histopathological injuries (Figure 5). In the ovaries of the control group, no morphological abnormalities were detected. They are composed of the ovary wall (ovisac) and several (7–8) ovary cords inside (Figure 5A–C). Each ovary cord is composed of numerous germline cells (oogonia, nurse cells, previtellogenic, and vitellogenic oocytes) with a regular distribution and typical architecture. Three principal zones corresponding to the successive stages of oogenesis can be distinguished along the long axis of the ovary cord (Figure 5A–C). The anterior zone is inhabited by undifferentiated germ cell cysts. The middle part was mostly occupied by growing oocytes (Figure 5A), while in the third part, degenerative germ cells were noted (Figure 5A–C).

Compared with those in the control group, exposure to PE-MPs at different concentrations led to an increase in damage severity in a concentration-dependent manner. The ovaries exposed to 1 µg/L of PE-MPs exhibited a progressive degradation of germline cyst integrity (Figure 5D–F). Moreover, in *E. johanssoni* ovaries treated with 10 µg/L of PE-MPs, we noted the loss of the typical organization of the ovary cord due to the dissolution and breakage of intercellular bridges between germ cells (Figure 5G–I). As illustrated in Figure 5J–O, significantly more abnormalities were observed in *E. johanssoni* gonads contaminated with 100 g/L or 1000 g/L of PE-MPs than in those in the control group, as revealed by massive necrosis and degeneration of germ cells (Figure 5J–O). Moreover, an increase in the number of degenerating oocytes was detected (Figure 5J–O). Indeed, some freely floating oocytes showed compromised morphology that was generally characterized by an irregular outline of the nuclear membrane in some areas (Figure 5O) and a less compact cytoplasm with large vacuolation. Additionally, we observed atrophied ovary cords showing a large lumen and degeneration of previtellogenic and vitellogenic oocytes due to necrosis.

### 3.2. Enzymatic Antioxidant Responses

**Figure 6:**
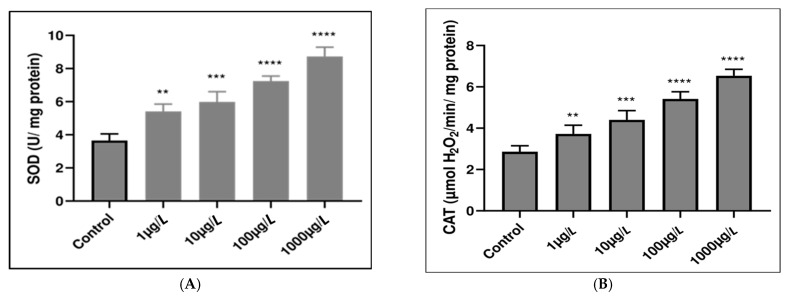

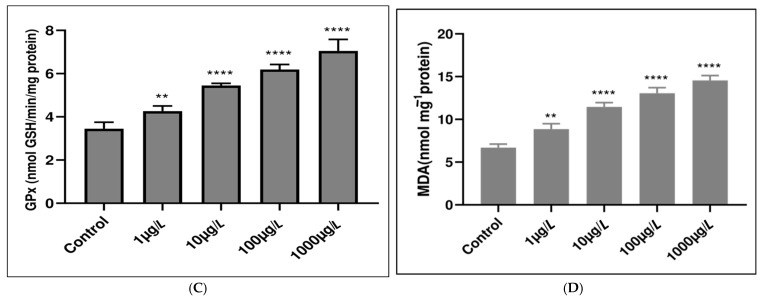
Effect of polyethylene microplastic (PE-MP) treatment on oxidative stress biomarkers in treated tissues—(**A**) superoxide dismutase (SOD) activity, (**B**) catalase (CAT) activity, and (**C**) glutathione peroxidase (GPx) activity—and on (**D**) malondialdehyde (MDA) levels in treated leeches. The values are expressed as the means ± SDs. ** *p* < 0.01; *** *p* < 0.001; **** *p* < 0.0001 indicate a significant difference from the control.

To detect the involvement of oxidative stress in polyethylene microplastic-induced damage in the freshwater leech *E. johanssoni*, the activities of the antioxidant enzymes SOD, CAT, and GPx were assayed. As shown in Figure 6, exposure to 1, 10, 100, or 1000 µg/L of PE-MPs substantially influenced antioxidant enzyme activities. SOD activity exhibited a significant increasing trend in a dose-dependent manner compared with that in the control group (*p* < 0.0001) (Figure 6A). CAT activity gradually increased in a dose-dependent manner (*p* < 0.001) at all PE-MP concentrations tested compared with that in the untreated group, as shown in Figure 6B. Additionally, Gpx activity exhibited a significant increasing trend in a dose-dependent manner in all the exposed leeches (*p* < 0.0001) compared with the control (Figure 6C).

### 3.3. Nonenzymatic Antioxidant Responses

Lipid peroxidation status was evaluated by measuring malondialdehyde (MDA) levels in the leech *E. johanssoni*, and the results are shown in Figure 6D. Exposure to 1, 10, 100, or 1000 µg/L of PE-MPs resulted in a perceptible increase in MDA levels in exposed leeches in a dose-dependent manner compared with those in the control (*p* < 0.0001).

### 3.4. Behavioral Alteration

**Figure 7:**
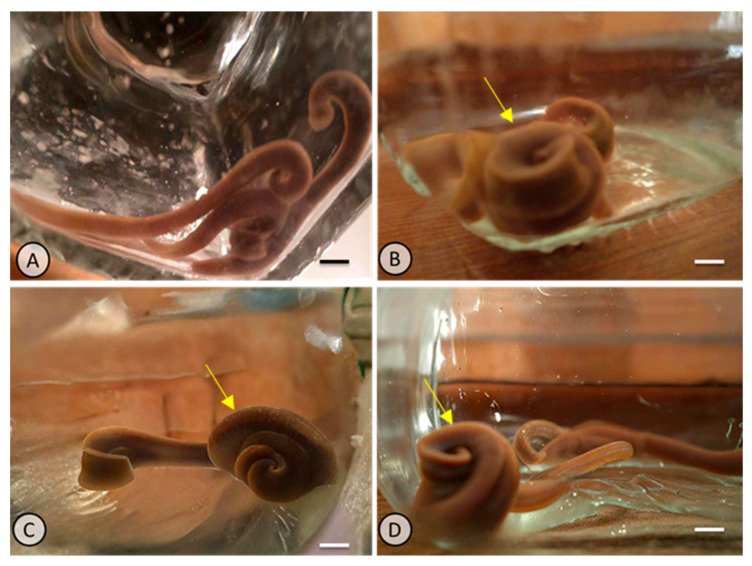
Morphological changes in *E. johanssoni* exposed to PE-MPs. (**A**–**D**): Changes in body shape of exposed specimens, including contraction and coiling (arrows), were noted. (**A**,**B**): Scale bar = 1 cm. (**C**): Scale bar = 0.8 cm. (**D**): Scale bar = 0.7 cm.

Several morphological transformations were observed in the exposed leeches (Figure 7A–D). These alterations encompass both changes in body shape and in mobility. Indeed, contractions of some muscles, abnormal positioning of suckers, coiling, contraction, and overall shortening of the leech’s body can occur. Moreover, swimming activity increased, likely indicating a significant impact on neurobehavioral responses.

## 4. Discussion

### 4.1. Microplastic-Induced Tissue Damage

The current study demonstrated that histopathological evidence supported our biochemical findings. Histopathology is increasingly recognized as a biomarker of environmental stress, with histopathological responses serving as valuable indicators of toxicity [58,59,60]. Microplastics (MPs) can cause tissue damage in aquatic organisms through several interrelated mechanisms. Their small size allows them to penetrate tissues, where they generate reactive oxygen species (ROS), leading to oxidative stress that damages cellular components. This oxidative stress can activate inflammatory pathways, resulting in inflammation and apoptosis. Additionally, the physical presence of MPs can disrupt cellular structures, further contributing to tissue damage. In our study, light microscopy examination of the *E. johanssoni* body wall, muscle, botryoidal cells, and ovaries revealed severe histological alterations.

### 4.2. Histological Analysis of the Body Wall and Botryoidal Tissue of Untreated and PE-MPs-Exposed Leeches

In untreated *Erpobdella johanssoni*, three distinct types of secretory cells were identified within the epidermis: type 1a (light-stained and pear-shaped cells), type 1b (dark-stained cells), and type 2 (large secretory cells). Only the latter cells reach the inner parts of the body wall, especially around the longitudinal muscles. Similarly, similar numbers of secretory cells were found in several freshwater and terrestrial leeches: *Erpobdella octoculata* and *Haemopis sanguisuga* [61], *H. sulukii* and *H. verbana* [49], and *Limnatis nilotica* [62] and *Haemadipsa zeylanica* [63]. Additionally, Ahmed and Rahemo [61] described two other non-secretory cells in their study on *Erpobdella octoculata*: pigment cells and supporting cells. Furthermore, these latter authors proposed that the pear-shaped cells, also referred to as type 1a in the present study, contain coarse granules crucial for the formation of the cocoon’s two opercula. In the case of the Glossiphoniide leech *Theromyzon tessulatum*, Seyers et al. [64] identified two types of non-secretory cells and four types of secretory cells within the epidermis, two of which were responsible for cocoon formation. Furthermore, according to [64], it was proposed that in *T. tessulatum*, the supporting cells seem to play a minor role in contributing to cocoon formation. However, Gorgees et al. [65] identified three types of glandular (secretory) cells and three types of non-glandular cells within the epidermis of lumbricid worms. Moreover, Morris [66], a study focusing on the clitellar epidermal cells of the red wiggler worm *Eisenia foetida* (Annelida: Oligochaeta), identified similar glandular cells, noting that two of these types are responsible for cocoon production. Notably, in this study, these two types of non-secretory cells (pigmented and supported) were not observed. However, it is crucial to emphasize that the destruction of these secretory cells may have a significant impact on cocoon formation.

In exposed leeches, various alterations in the body wall have been observed. The morphology, and likely the functional state of the secretory cells, changes from rounded, inactive cells, which are characteristic of the control group, to elongated, pear-shaped, active secretory cells in treated leeches. It seems that the rounded cells, which lack contact with the body wall surface, indicate a state of inactivity, likely associated with reduced mucus production. In contrast, elongated cells, with their enlarged appearance, actively participate in mucus production and secretion. The pear-shaped and elongated morphology of these cells may play a facilitating role, potentially accommodating ductules for improved mucus release efficiency and establishing a connection to the body wall surface. These cells adopt an elongated, pear-shaped form, a characteristic feature enabling them to extend toward the epithelial layer, thus enhancing the secretion of mucus into the surrounding environment.

Similar reactions in the body wall have been noted in *Hirudo verbana* exposed to polypropylene (PP) micro- and nanoplastics [67]. Indeed, it is widely accepted that the first line of defense against potentially harmful substances, as perceived by animals, is represented by the production of mucus by secretory cells [49,67,68]. Accordingly, Baranzini et al. [67] noted that in untreated *H. verbana*, the mucus cells are typically rounded in shape and remain inactive. They are located beneath the epithelium and among the muscle fibers. However, following exposure to PP microplastics and nanoplastics for 1 h, 6 h, and 1 week, both types of mucus cells exhibit elongation and thus an active form. However, by 1 month, these cells are once again visible in both active and inactive states, indicating that the restoration of secretory cells is required for secretion and facilitates new waves of mucus production. Furthermore, in *E. johanssoni*, variations in the number of secretory cells have also been observed. Specifically, in the studied leeches exposed to PE-MPs, type 2 inner secretory cells migrated to the epidermis to reinforce mucus secretion, leading to an increase in the number of elongated, active mucus cells. Our findings align with those of [68], who notably observed a pronounced proliferation of mucus cells in the epidermis of *L. nilotica* exposed to cadmium. A similar response was documented in *H. verbana* by the authors of [67], who suggested that the number of mucus cells in the inner muscle layer was reduced, indicating migration toward the subepithelial area. In addition to affecting cell morphology and number, other reactions have been observed, including cuticle detachment, degeneration, and vacuolization of epithelial cells, along with an increase in the number of blood vessels. Similar findings were reported in *Limnatis nilotica* exposed to cadmium in [68] and in *Hirudo verbana* exposed to copper and polypropylene micro- and nanoplastics, respectively [67,69]. Additionally, degenerated fibroblasts, decreased cuticular folds, and body undulations were noted in *H. verbana* exposed to copper [69]. In the present investigation, while untreated *E. johanssoni* appeared avascular, interestingly, the number of blood vessels increased in exposed leeches, indicating prompt activation and an early reaction to the presence of PE-MPs. Similar findings were noted in *H. verbana*, where over two months, there was a continuous increase in new blood vessels and recruited cells. Hematopoietic precursor cells from botryoidal tissues move through vessels, spreading across the leech body wall. These cells then transform into macrophages, signaling an early inflammatory response to plastic particle uptake [67]. Furthermore, it was demonstrated that botryoidal tissue cells change their shape and function, thus cooperating with new vessel formation [70,71]. The analysis of the botryoidal tissue of the investigated species confirmed these findings. In fact, in the control group, botryoidal cells were not activated and formed stable cords of clustered, rounded cells. However, in treated animals, relevant morphological changes were observed. Indeed, solid cellular cords were observed to develop a lumen. This transformation was facilitated by the ability of botryoidal tissue cells to shape a luminal cavity. Notably, these cells underwent significant shape modifications, including thinning, flattening, and tapering, enabling vessels to increase both their diameter and length. Furthermore, in *Erpobdella johanssoni*, the cavity of the new vessel consisted of a central lumen lined by botryoidal tissue cells. Thus, our results confirm the notion that botryoidal tissue plays a role in angiogenesis [70]. The observed morphological changes in the studied *E. johanssoni*, along with their corresponding responses, could be interpreted as protective adaptations aimed at minimizing the detrimental effects of pollutants. Furthermore, these alterations might have the potential to interfere with the regular functioning of the affected organ.

Girardello et al. [72] demonstrated that small particles in medicinal leeches are capable of traversing both the cuticle and the epithelial layers, thereby penetrating the body wall and accumulating in the connective tissue. This observation was further confirmed by fluorescence analyses in *Hirudo verbana* by the authors of [67]. In fact, various plastic particles are situated beneath the epithelium and surrounding muscle fibers and are dispersed within the leech’s connective tissue. These findings may affect the functionality of muscle fibers.

### 4.3. Histological Analysis of Muscle Cells and Its Possible Relationship with Behavioral Alterations

The body wall muscles inside the internal dermis play a crucial role in the locomotion and swimming activity of aquatic leeches [73]. In the studied leech, these muscles consist of several layers, including a relatively thin outer layer of circular muscles, a thin layer of oblique muscles, a dorsoventral muscle, and a thick inner layer of longitudinal muscles. This muscle arrangement was also observed by the authors of [74] in their study on *Erpobdella octoculata*, particularly in the clitellar region. Furthermore, it appears that each type of muscle plays a distinct role in both movement and changes in body shape. In fact, the authors of [75] found that when moving on land, the circular and longitudinal muscles show rhythmic activity, while the dorsoventral muscles remain inactive. However, in water, the dorsoventral muscles contract continuously while the circular muscles relax, and waves of contraction move alternately along the ventral and dorsal bands of longitudinal muscles [75]. In treated leeches, alterations in the morphology of muscle cells, particularly longitudinal fibers, have a noticeable impact on muscle fibers within the studied species. Our research results align with those of prior studies, such as [69], which investigated the effects of copper on the body wall, muscle fibers, and fibroblasts of the medicinal leech *H. verbana*. On the other hand, when investigating behavioral alterations in *E. johanssoni*, changes in mobility, body posture, and shape were observed. Therefore, these initial responses documented in our study could serve as a rapid method for assessing water pollution levels and may predict harmful consequences of prolonged exposure to toxic substances.

Similarly, medicinal leeches (*Hirudo medicinalis*) bred in controlled environments exhibited distinct behavioral responses when exposed to three different conditions: (1) water sourced from Lake Drukshiai, the cooling reservoir of the Ignalina Nuclear Power Plant; (2) sediments from the Nemunas River; and (3) a solution containing a model mixture of heavy metals (HMMM). These responses encompass alterations in mobility and shifts in body morphology. Moreover, feeding activity and avoidance responses were also noted [76]. Nevertheless, the body wall of the leech comprises four robust layers of muscles; thus, the contraction of certain muscles results in visible alterations in the shape of the animal. These observable changes in body shape were suggested to indicate disruptions in enzyme activity within the central nervous system of *Hirudo verbana* [77]. In fact, decreased inactivation of acetylcholine, a neurotransmitter at the neuromuscular junction, triggers the continuous depolarization of muscles, leading to constant muscle contraction in both *H. verbana* and *H. medicinalis* [76,77]. In the present investigation, acetylcholinesterase activity was not measured, but we strongly suggest that the first reaction to changes in the body shape of the leech suggested disruptions and perturbations in the activity of this enzyme within its central nervous system. Moreover, alterations in the morphology of the muscle fibers of the studied leeches evidently affected the locomotor cycle. Indeed, muscle cells are crucial for body shape changes and interactions with the environment, allowing leeches to attach, move, and detach effectively.

In a similar vein, Zink et al. [78] explored how microplastics, cadmium, and their combination affected the freshwater leech *Nephelopsis obscura* over a 21-day exposure period. Their findings align with our observations, as they demonstrated that microplastic exposure led to decreased serotonin levels which, in turn, disrupted feeding behaviors. Given serotonin’s pivotal role in leech physiology—regulating locomotion by stimulating muscle contractions for peristaltic movements, enhancing feeding through pharyngeal muscle activation, and acting as a neuromodulator in reflexes and motor coordination [79]—these disturbances highlight a broader pattern of neuromuscular disruption in annelids exposed to environmental stressors.

### 4.4. Histological Analysis of the Female Gonad

The morphology of untreated ovaries conforms to the results of a detailed study conducted on the same species by [45,80]. However, the present investigation revealed that prolonged exposure to polystyrene microplastics had a negative impact on the female gonads of the examined leeches. Potential impacts were noticeable even at relevant concentrations (1, 10, and 100 mg/L), with more pronounced effects observed at higher theoretical concentrations (1000 mg/L) during a 7-day exposure period. The ovaries of exposed leeches displayed numerous changes, including disruptions in the organization of the ovary cord caused by vacuolation and disconnection between germ cells and the cytophore. Additionally, degeneration and necrosis were observed in the freely floating vitellogenic oocytes. Histopathological examination of gonads following exposure to microplastics reveals significant impacts on the reproductive organs of various aquatic species. Studies conducted separately on zebra fish (*Danio rerio*) by [81] and on the catfish *Clarias gariepinus* by [82] have shown that exposure to polystyrene microplastics can trigger molecular responses, induce histological changes, and increase apoptosis levels in the gonads of male fish. These changes lead to alterations in gonadal cell structure, developmental delays, and disruptions in both gonadal maturity levels and gonadosomatic index values. Damage to gonadal tissue and decreased viability of gametes represent some of the most severe outcomes of water pollution. These effects lead to lower reproductive success rates and reduced organismal fitness [83]. Furthermore, several studies have demonstrated that the presence of microplastics in gonadal tissue has a negative impact on the fecundity and fertility of organisms [84]. As highlighted by [85], the ingestion of microplastics during gametogenesis negatively impacts reproduction in offspring. The repercussions of microplastic contamination on the reproductive health of aquatic species highlight the critical need to comprehend and mitigate the adverse effects of microplastics on the environment and aquatic ecosystems.

### 4.5. Microplastic Impact on Antioxidant System Functionality

Research on the induction of oxidative stress by MPs typically involves two main aspects: antioxidative defenses, such as superoxide dismutase (SOD), catalase (CAT), glutathione peroxidase (GSH-Px), glutathione S-transferase (GST), and glutathione reductase (GR) enzymes, and oxidative damage, including lipid peroxidation (LPO) [86,87]. Assessing oxidative stress biomarkers provides key insights into general toxicity [88,89]. Antioxidant enzymes play a crucial role in protecting cells against oxidative damage induced by microplastics by neutralizing reactive oxygen species (ROS) and mitigating intracellular imbalances [67]. The disparity between ROS production and antioxidant activity enhances enzyme responses, notably SOD, CAT, and GPx [90]. SOD catalyzes the dismutation of the superoxide anion (O_2_^−•^) into hydrogen peroxide (H_2_O_2_) and oxygen (O_2_), while CAT and GPx subsequently reduce H_2_O_2_ to water [68]. These enzymes act as key defenses against oxidative stress via the Fenton reaction [91,92].

Lipid peroxidation, a consequence of ROS targeting cellular lipids, leads to the formation of malondialdehyde (MDA), a key biomarker for oxidative damage [93,94]. MDA is produced from the breakdown of polyunsaturated fatty acids and serves as an indicator of oxidative stress and tissue damage [68,95].

In the present study, the selection of SOD, CAT, and GPx was based on their pivotal roles in the primary antioxidant defense system of aerobic organisms. Further, these enzymes are integral to mitigating oxidative stress induced by environmental pollutants such as microplastics (MPs). In the present study, the antioxidant enzymatic activities of CAT, SOD, and GPx increased after 7 days of exposure to PE-MPs. This increase was likely a defensive response aimed at combating oxidative stress and the generation of reactive oxygen species (ROS). Indeed, our data indicate an increase in SOD activity, suggesting that *E. johanssoni* has an enhanced ability to eliminate O_2_-. Furthermore, the increase in CAT activity suggested that it could be triggered by the accumulation of H_2_O_2_ originating from the metabolites of SOD and other biotransformation processes. The increase in GPx activity might be explained by the crucial role this biomarker plays in eliminating H_2_O_2_ within the studied antioxidant defense system of leeches. Additionally, in our study, the MDA level increased significantly in all PE-MP-treated leeches, demonstrating a significant positive correlation with the concentration of PE-MPs. Such an increase in MDA levels could undermine both membrane integrity and functionality. The increase in MDA levels detected in stressed leeches is indicative of the production of reactive oxygen species and the extent of oxidative stress. Hence, the significant increase in MDA is widely acknowledged as a primary oxidative byproduct resulting from the presence of polyunsaturated fatty acids within biological membranes [68].

A similar increase in GST and SOD activities was also detected in the medicinal leech *Hirudo verbana* after 7 days of exposure to polypropylene micronanoplastics (PP-MNPs) [67]. Furthermore, polyethylene microplastics were reported to induce oxidative stress in *Mytilus galloprovincialis* by decreasing CAT and glutathione S-transferase (GST) activities, as well as lipid peroxidation (LPO) levels, after 14 days of exposure [87]. Similarly, our results correlated with those found in the farmed Pacific white shrimp *Penaeus vannamei*. It appears that microplastics can indeed affect the activity of oxidase enzymes and the concentrations of MDA in this species. Additionally, similar observations have been reported, indicating that exposure to polystyrene (PS) was accompanied by decreased CAT enzyme activity and elevated MDA content of lipid peroxides in San Francisco brine shrimp (*Artemia franciscana*) [96]. Moreover, the MDA content of thick-shell Coruscus mussels (*Mytilus coruscus*) has been observed to increase under the influence of PS [94]. We therefore suggested the usefulness of biomarkers in evaluating the impacts of commonly produced and prevalent polymers on nontarget freshwater organisms. We also performed histopathological analysis of the body wall, muscle cells, botryoidal cells, and ovaries. Accordingly, tissue alterations serve as a reliable indicator for conducting ecological risk assessments and various ecotoxicological studies.

## 5. Conclusions

In this study, we have demonstrated, for the first time, the toxicity of polyethylene microplastics (PE-MPs) to the freshwater leech *Erpobdella johanssoni*. Our results show a clear increase in oxidative stress, with higher PE-MPs concentrations, even at environmentally relevant levels. In addition, histopathological alterations observed in this species provide critical information on the effects of PE-MPs, suggesting that these changes could serve as useful biomarkers for future ecotoxicological studies of microplastics.

This research also highlights the potential of leech species as bioindicators in aquatic environments, owing to their direct exposure to pollutants, ease of maintenance, and well-documented physiological responses. The data provided in this study can serve as a basis for future monitoring programs designed to assess the impact of emerging contaminants on freshwater ecosystems and their nontarget organisms.

However, there are some limitations to this study. It focused exclusively on polyethylene microplastics, and future research should explore the toxicity of other types of microplastics, such as tire wear particles and biodegradable microplastics, to provide a broader understanding of their effects on freshwater species. Additionally, it is crucial to investigate the long-term and multigenerational impacts of microplastics on freshwater organisms, as these effects are often not apparent in short-term studies.

To better understand the broader ecological consequences of microplastic pollution, further research is needed to assess how microplastics accumulate in freshwater environments over time and affect ecosystem functions. There is also a need to explore the interactions between microplastics and other pollutants present in aquatic systems, which may exacerbate their toxic effects. As the global production and disposal of plastic materials continue to increase, a long-term approach to managing microplastic pollution is essential. This approach should focus not only on the immediate effects on individual species but also on how microplastics may alter the dynamics and biodiversity of ecosystems in the future.

## Figures and Tables

**Table 1:** Summary of microplastic concentrations in several freshwater environments.

Study Location	Matrix Type	Average Concentration	Size Range (μm)	Dominant Polymer Types	Reference
Seine River, France	Water	100.6 ± 99.9 fibers/m^3^	50–5000	PET	[29]
Teltow Canal, Germany	Water	7.86 ± 7.26 MPs/L	450–5000	PE, PP	[29]
Carpathian Basin, Hungary	Water	3.52–32.05 particles/m^3^	100–2000	PP, PE, PS	[30]
Lake Bolsena, Italy	Water	0.82–4.42 MPs/m^3^	>300	Not specified	[29]
River Thames, UK	Sediment	33.2 ± 16.1 particles/100 g	1000–5000	PP, PET	[31]
Jedara Stream, Tunisia	Sediment	6920 ± 395.98 items/kg	200–5000	PP, PE	[28]
Khima Stream, Tunisia	Sediment	2340 ± 227.15 items/kg	200–5000	PP, PE	[28]

## Data Availability

The data that support the findings of this study are available from the corresponding author upon reasonable request.

## Abbreviations

The following abbreviations are used in this manuscript:

PE-MPs	Polyethylene microplastics
MPs	Microplastics
MDA	Malondialdehyde
CAT	Catalase
SOD	Superoxide dismutase
GPx	Glutathione peroxidase
